# Inhibition of adenovirus transport from the endosome to the cell nucleus by rotenone

**DOI:** 10.3389/fphar.2023.1293296

**Published:** 2024-01-11

**Authors:** María Balsera-Manzanero, Francesca Ghirga, Ana Ruiz-Molina, Mattia Mori, Jerónimo Pachón, Bruno Botta, Elisa Cordero, Deborah Quaglio, Javier Sánchez-Céspedes

**Affiliations:** ^1^ Unidad Clínica de Enfermedades Infecciosas, Microbiología y Parasitología, Instituto de Biomedicina de Sevilla (IBiS), Hospital Universitario Virgen del Rocío/CSIC/Universidad de Sevilla, Sevilla, Spain; ^2^ Department of Chemistry and Technology of Drugs, Sapienza University of Rome, Rome, Italy; ^3^ Department of Biotechnology, Chemistry and Pharmacy, University of Siena, Siena, Italy; ^4^ Instituto de Biomedicina de Sevilla (IBiS), Hospitales Universitarios Virgen del Rocío y Virgen Macarena/CSIC/Universidad de Sevilla, Sevilla, Spain; ^5^ Departamento de Medicina, Facultad de Medicina, Universidad de Sevilla, Sevilla, Spain; ^6^ CIBERINFEC, ISCIII—CIBER de Enfermedades Infecciosas, Instituto de Salud Carlos III, Madrid, Spain

**Keywords:** rotenoids, adenovirus infection, antiviral compound, cytomegalovirus, microtubular polymerization

## Abstract

Regardless of the clinical impact of human adenovirus (HAdV) infections in the healthy population and its high morbidity in immunosuppressed patients, a specific treatment is still not yet available. In this study, we screened the CM1407 COST Action’s chemical library, comprising 1,233 natural products to identify compounds that restrict HAdV infection. Among them, we identified rotenolone, a compound that significantly inhibited HAdV infection. Next, we selected four isoflavonoid-type compounds (e.g., rotenone, deguelin, millettone, and tephrosin), namely rotenoids, structurally related to rotenolone in order to evaluate and characterized *in vitro* their antiviral activities against HAdV and human cytomegalovirus (HCMV). Their IC_50_ values for HAdV ranged from 0.0039 µM for rotenone to 0.07 µM for tephrosin, with selective indices ranging from 164.1 for rotenone to 2,429.3 for deguelin. In addition, the inhibition of HCMV replication ranged from 50% to 92.1% at twice the IC_50_ concentrations obtained in the plaque assay for each compound against HAdV. Our results indicated that the mechanisms of action of rotenolone, deguelin, and tephrosin involve the late stages of the HAdV replication cycle. However, the antiviral mechanism of action of rotenone appears to involve the alteration of the microtubular polymerization, which prevents HAdV particles from reaching the nuclear membrane of the cell. These isoflavonoid-type compounds exert high antiviral activity against HAdV at nanomolar concentrations, and can be considered strong hit candidates for the development of a new class of broad-spectrum antiviral drugs.

## 1 Introduction

Human adenoviruses (HAdVs) are non-enveloped, double-stranded DNA viruses that belong to the genus Mastadenovirus of the Adenoviridae family (Mennechet et al., 2019). HAdV infections can be a major problem in hematopoietic stem cell transplant and solid organ transplant recipients, especially in pediatric units, where they can cause severe pneumonia, diarrhea, hemorrhagic colitis, hepatitis, and hemorrhagic cystitis, and disseminated HAdV disease (in up to 10% of the cases) (Lion, 2014). In these patient populations, co-infection with human cytomegalovirus (HCMV), *Aspergillus*, or bacteria occurs frequently and may contribute to the poor outcomes associated with HAdV disease, with significant mortality rates (26%–80%) (Wold and Toth, 2015; Ison and Hayden, 2016). Moreover, the clinical impact of HAdV, which is one of the most frequent viral etiologies of community-acquired pneumonia, has been confirmed in the recent years (Zhang et al., 2020).

Regardless of the high clinical impact of HAdV infections in these populations, no specific treatment is available for HAdV infections, and clinical management is currently based on existing therapies, including the administration of immunoglobulins or the use of broad-spectrum antiviral drugs. Currently, the drug of choice for the clinical treatment of HAdV infections is cidofovir (CDV), although its use has several disadvantages, including low bioavailability after oral administration and nephrotoxicity (Lion, 2014; Schaar et al., 2016; Grimley et al., 2017). Thus, developing antiviral drugs with proven activity against HAdVs is urgently required.

Recently, our research team has focused on the discovery and development of anti-HAdV agents (Sanchez-Cespedes et al., 2016; Marrugal-Lorenzo et al., 2018; Marrugal-Lorenzo et al., 2019; Aguilar-Guisado et al., 2020; Mazzotta et al., 2020a; Xu et al., 2020a; Xu et al., 2020b). As a part of our continuous effort in this research line, we joined in 2017 the COST Action CM1407 “Challenging Organic Syntheses Inspired by Nature-From Natural Products Chemistry to Drug Discovery,” a European cooperation network aimed at discovering new naturally occurring small molecules of pharmaceutical relevance. Over the years, a large CM1407 Action library was created, resulting in a strategy for finding new hits and designing new chemical compounds. In this context, the CM1407 Action’s chemical library, comprising 1,233 natural products, was screened against HAdV. Among these, rotenolone, a rotenoid compound, exhibited the most promising antiviral characteristics.

**FIGURE 1:**
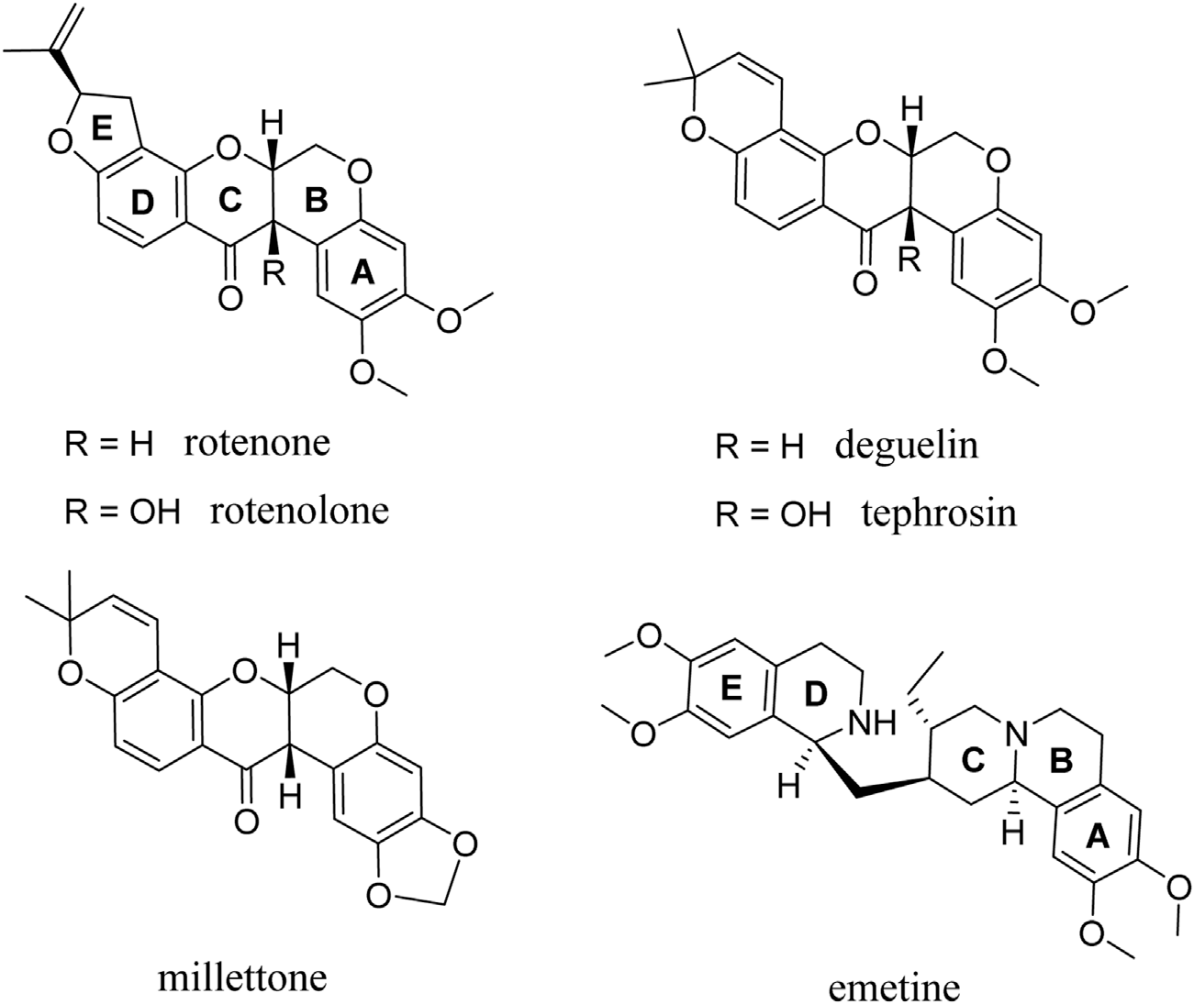
Chemical structure of the rotenolone-related compounds evaluated.

Rotenoids are a class of isoflavonoids widely distributed in the Fabaceae family. They are traditionally used as pesticides and insecticides and present moderate toxicity in animals, as illustrated by the estimated lethal dose of 300–500 mg/kg for rotenone (Ray et al., 1991; Vats, 2018; Vats and Mehre, 2020). Additionally, rotenone is also commonly used to model Parkinson’s disease in rats and requires large systemic doses of rotenone (>2–3 mg/kg/day) for long periods of time (>3 weeks) (Betarbet et al., 2000; Bove et al., 2005). Interestingly, rotenolone has structural similarities with emetine (Figure 1), a natural alkaloid derived from the ipecac root and an FDA-approved drug for the treatment of amebiasis, which has previously been reported to have significant broad-spectrum antiviral activity (Mukhopadhyay et al., 2016; Khandelwal et al., 2017; MacGibeny et al., 2018; Yang et al., 2018; Wang et al., 2020). In general, emetine is characterized by a complex molecular structure comprising of a monoterpenoid-tetrahydroisoquinoline skeleton with five interconnected rings labeled A-E (Figure 1). Emetine possesses a high degree of structural flexibility because of the presence of a methylene bridge connecting rings D and E to rings A, B, and C. Although rotenoids have a five-ring fused structure featuring a thermodynamically stable cis-B/C ring, they possess good flexibility with a preferentially bent structural conformation at the face of contact between the B/C ring fusion (Figure 1). Altogether, these structural features, along with the existence of electron-donating groups on both rings A and E of emetine and the selected rotenoids, impart similar spatial orientations and electronic properties.

Rotenoids have been used as a lead molecules in the synthesis of several analogues and have been reported to exhibit various pharmacological properties, making them ideal for structural modification and structure-activity relationship analysis (Russell et al., 2018; Xu et al., 2018). In fact, as already shown by Takatsuki *et al.* (Takatsuki et al., 1969), some of the rotenoids studied here already showed antiviral activity against Newcastle disease and herpes simplex viruses, although their potential mechanism of action was not explored. Based on our findings, four rotenolone-related compounds featuring the same cis-fused tetrahydrochromeno[3,4-*b*]chromene nucleus (B and C rings) and different patterns of substitution in all rings (A-D) were chosen and tested against HAdV. Herein, we present a detailed evaluation of the antiviral activity of rotenoids and the characterization of their potential mechanisms of action (Figure 1).

## 2 Materials and methods

### 2.1 Chemistry

All rotenoids are known structures that belong to the *in-house* library of natural products available at the Organic Chemistry Laboratory of the Department of Chemistry and Technology of Drugs of Sapienza University of Rome, Italy. The chemical identities of the compounds were assessed by re-running the NMR experiments and found to be in agreement with the literature data reported below for each compound. The purity of all compounds, as determined by reversed-phase high performance liquid chromatography (HPLC), was always >95%.

Rotenolone or (2R,6S,6aR,12aS)-8,9-dimethoxy-2-(prop-1-en-2-yl)-1,2,6,6a,12,12a-hexahydrochromeno[3,4-b]furo[2,3-h]chromen-6-ol displayed NMR spectra identical to those reported in the literature (Russell et al., 2020). Rotenone or (2R,6aS,12aS)-8,9-dimethoxy-2-(prop-1-en-2-yl)-1,2,12,12a-tetrahydrochromeno[3,4-b]furo[2,3-*h*]chromen-6-(6a*H*)-one was purchased from TCI (Tokyo Chemical Industry) (CAS: 83-79-4, Tokyo, Japan) and used without further purification. Deguelin or (7aS,13aS)-13,13a-Dihydro-9,10-dimethoxy-3,3-dimethyl-3H-bis (Mennechet et al., 2019)benzopyrano[3,4-b:6′,5′-e]pyran-7-(7aH)-one was purchased from Sigma-Aldrich (CAS: 522-17-8, St. Louis, MO, USA) and used without further purification. Tephrosin or hydroxydeguelin was purchased from Sigma-Aldrich (CAS: 76-80-2, St. Louis, MO, USA) and used without further purification. Millettone displayed NMR spectra identical to those reported previously (Yenesew et al., 1998).

All compounds were dissolved and diluted in dimethyl sulfoxide (DMSO; Sigma-Aldrich) and maintained at −20°C until further use.

### 2.2 Cell lines and virus strains

Human A549 cell line was obtained from the American Type Culture Collection (ATCC, Manassas, VA, USA). The 293β5 stable cell line overexpressing the human β5 integrin subunit was provided by Dr. Glen Nemerow (Nguyen et al., 2010). The cell lines were propagated in Dulbecco’s modified Eagle medium (DMEM; Life Technologies/Thermo Fisher Scientific, MA, USA) supplemented with 10% fetal bovine serum (FBS) (Omega Scientific, Tarzana, CA), 10 mM HEPES, 4 mM l-glutamine, 100 units/mL penicillin, 100 μg/mL streptomycin, and 0.1 mM non-essential amino acids (complete DMEM).

Wild-type HAdV5 and HCMV were obtained from the ATCC (references VR-5 and AD169, respectively). The HAdV5-GFP used in this study is a replication-defective virus containing a CMV promoter-driven enhanced green fluorescent protein (eGFP) reporter gene cassette in place of the E1/E3 regions (Nepomuceno et al., 2007). HAdV-RFP is a wild-type HAdV5 reporter construct containing the red fluorescent protein (RFP) gene under the regulation of the HAdV MLP; thus, RFP is expressed following active viral DNA replication. HAdV-RFP was provided by Dr. Robin Parks (Ottawa Hospital Research Institute) (Saha et al., 2019). AdV2*ts*1, an endosome penetration-defective HAdV mutant, was provided by Dr. Glen Nemerow (The Scripps Research Institute, CA, USA) (Weber, 1976; Sanchez-Cespedes et al., 2014). HAdV were propagated in 293β5 cells and isolated from the cellular lysate using cesium chloride density centrifugation. Virus concentration, in mg/mL, was calculated using the Bio-Rad Protein Assay (Bio-Rad Laboratories) and converted to virus particle (vp) concentration (1 µg = 4 × 10^9^ virions) (Smith and Nemerow, 2008).

### 2.3 Screening of COST Action’s chemical libraries

An initial rapid screening was performed using human A549 cells (3 × 10^5^ cells/well) infected with HAdV5-GFP with a multiplicity of infection (MOI) of 2,000 vp/cell in the presence of 50 μM of the candidate antiviral compounds. Briefly, a standard infection curve was generated in parallel by infecting cells in the absence of compounds using serial two-fold dilutions of virus. Cells, virus and compounds were incubated for 48 h at 37°C and 5% CO_2_. Infection, as measured by HAdV5-mediated GFP expression, was analyzed using a Typhoon 9410 imager (GE Healthcare Life Sciences), and quantified with ImageQuantTL (GE Healthcare Life Sciences). All reactions were done in triplicate.

### 2.4 HAdV plaque assay

Compounds were tested using low MOI infections (0.06 vp/cell), initially at concentrations of 10 μM and subsequently in a dose-response assay ranging from 10 μM to 2.44 nM in a plaque assay. Briefly, 293β5 cells were seeded in 6-well plates at a density of 1 × 10^6^ cells per well in duplicate for each condition. When the cells reached 80%–90% confluency, they were infected with HAdV5-GFP and rocked for 2 h at 37°C. After the incubation, the inoculum was removed and the cells were washed once with PBS. Cells were then carefully overlaid with equal parts of 4 mL/well 1.6% (water/vol) Difco Agar Noble (Becton, Dickinson & Co., Sparks, MD, USA) and 2× Eagle’s Minimum Essential Medium (EMEM; BioWhittaker) supplemented with 2×penicillin/streptomycin, 2× L-glutamine, and 10% FBS. The mixture also contained the compounds in the concentration ranges described above. Following incubation for 7 days at 37°C, plates were scanned with a Typhoon FLA 9000 imager (GE Healthcare Life Sciences) and the formed plaques were quantified with ImageJ (Schneider et al., 2012). Plaque counting was performed to calculate the 50% inhibitory concentration (IC_50_) of each compound using the statistical software GraphPad Prism 6.

### 2.5 HAdV DNA quantification by qPCR

For DNA quantification, A549 cells (1.5 × 10^5^ cells/well in a 24-well plate) were incubated for 24 h in 500 μL of complete DMEM and were infected with wild-type HAdV5 (MOI 100 vp/cell) after observing more than 90% of confluency. Infected cells were incubated for 24 h at 37°C in 500 μL of either complete DMEM containing 10-fold IC_50_ concentration of the compounds obtained in the plaque assay or the same volume of DMSO (positive control). All samples were prepared in duplicate. After 24 h of incubation at 37°C, DNA was purified from the cell lysate with the E.Z.N.A.^®^ Tissue DNA Kit (Omega Bio-tek, Norcross, GA) following the manufacturer’s instructions. PCR conditions, primers, and probes were as described by Henke-Gendo et al. (2012). Human glyceraldehyde-3-phosphate dehydrogenase (GAPDH) gene was used as an internal control. GAPDH oligonucleotide sequences and conditions were as previously described by Rivera et al. (2004).

For quantification, gene fragments from hexons, and GAPDH were cloned into the pGEM-T Easy vector (Promega) and known concentrations of the template were used to generate a standard curve in parallel for each experiment, as previously reported (Sanchez-Cespedes et al., 2016). All assays were performed in thermal cycler LightCycler^®^ 96 System (Roche).

### 2.6 Nuclear-associated HAdV genomes

Nuclear delivery of HAdV genomes was assessed using qPCR following nuclear isolation from infected cells. Briefly, 1 × 10^6^ A549 cells in 6-well plates were infected either with wild-type HAdV5 at an MOI of 2,000 vp/cell in the presence of a 10-fold IC_50_ concentration of the compounds obtained in the plaque assay or the same volume of DMSO as the positive control. Forty-five minutes after infection, the A549 cells were trypsinized, collected, and washed twice with PBS. The cell pellet was resuspended in 500 μL of 1×hypotonic buffer (20 mM Tris-HCl pH 7.4, 10 mM NaCl, 3 mM MgCl_2_) and incubated for 15 min at 4°C. Then, 25 μL of NP-40 was added and the samples were vortexed. The homogenates were centrifuged for 10 min at 835 g at 4°C. Following the removal of the cytoplasmic fraction (supernatant), HAdV DNA was isolated from the nuclear (pellet) and cytoplasmic fractions using the E.Z.N.A.^®^ Tissue DNA Kit (Omega Bio-tek, Norcross, GA).

### 2.7 HAdV time of addition assay

The anti-HAdV effect of these natural compounds at different time-points was measured through a time-curve assay using 293β5 cells (3 × 10^4^ cells/well in Corning black wall, clear bottom 96-well plates) infected with HAdV5-GFP (MOI 2,000 vp/cell) in the presence of 100 μL of complete DMEM containing 10-fold IC_50_ concentration of each comound obtained in the plaque assay for each compound or the same volume of DMSO (positive control). A standard infection curve was generated in parallel by infecting cells in the absence of compounds using 2-fold serial dilutions of the virus from an MOI of 2,000 vp/cell in order to extrapolate the GFP resulting from each molecule with its corresponding MOI.

Parallel samples of HAdV5 were incubated with or without the selected compounds on ice for 1 h. HAdV5 was then added to 293β5 cells and incubated at 37°C. Compounds were then added at the indicated time points until the last time point at 2 h. Then, the cells were incubated for an additional 48 h at 37°C and 5% CO_2_ before being analyzed for GFP expression using a Typhoon 9410 imager (GE Healthcare Life Sciences) and ImageQuantTL (GE Healthcare Life Sciences), as we described earlier.

### 2.8 Cytotoxicity assay

The cytotoxicity of the molecules was analyzed using the commercial kit AlamarBlue^®^ (Invitrogen, Ref. DAL1025) according to the manufacturer’s instructions. Briefly, A549 cells were seeded at a density of 5 × 10^3^ cells per well in 96-well plates, and decreasing serial dilutions of each molecule were diluted in 100 μL of DMEM starting at 200 μM. The cells were then incubated at 37°C for 48 h following the manufacturer’s protocol. The cytotoxic concentration 50% (CC_50_) value was obtained using the statistical software GraphPad Prism 6. The assay was performed in duplicate.

### 2.9 HAdV yield reduction assay

A549 cells (1.5 × 10^5^ cells/well in a 24-well plate) were incubated 24 h in 500 μL of complete DMEM and they were infected with wild-type HAdV5 (MOI 100 vp/cell) when more than 90% of confluency was observed. Infected cells were incubated for 48 h at 37°C in 500 μL of either complete DMEM containing 10-fold IC_50_ concentration of compounds obtained in the plaque assay or the same volume of DMSO (positive control). After 48 h, the cells were harvested and subjected to three rounds of freeze/thaw. Serial dilutions of the clarified lysates were titrated against A549 cells (3 × 10^4^ cells/well in a 96-well plate) and TCID_50_ values were calculated using an end-point dilution method (Reed and Muench, 1938).

### 2.10 HAdV-mediated endosome disruption

A549 cells (2 × 10^4^ cells/well) were incubated in DMEM without cysteine or methionine supplemented with 10% dialyzed FBS [DMEM(−)] in black 96-well plates for 1 h prior to infection. Three-fold serial dilutions (0.45 ng–1,000 ng) of HAdV5, or AdV2*ts*1 were preincubated with cells in the presence of 10-fold IC_50_ concentration of the compounds obtained in the plaque assay or the same volume of DMSO for positive control for 1 h. The medium was then removed and replaced with 50 μL DMEM(−) containing 0.1 mg/ml α-sarcin (Santa Cruz Biotechnology, Dallas, Texas, USA) and drug mixtures. After 1 h at 37°C, the Click-iT L-homopropargylglycine (HPG) Alexa Fluor 488 Protein Synthesis Assay Kits (Invitrogen) was used to analyse protein synthesis according to the manufacturer’s instructions. The incorporation of the amino acid analogue of methionine HPG containing Alexa Fluor 488 azide was measured using a Typhoon 9410 imager (GE Healthcare Life Sciences) and calculated by subtracting the background level of the control well containing HPG and α-sarcin but not the virus (100% incorporation).

### 2.11 Evaluation of the p53/MDM2 complex

We assayed the capacity of these molecules to disrupt the *p53*/MDM2 interaction after infection with HAdV, as proposed for the anti-HCMV activity of emetine (Mukhopadhyay et al., 2016). A549 cells were seeded at a density of 1.5 × 10^5^ cells per well in a 24-well plate at a high density and 3.75 × 10^4^ cells per well at a low density. After 24 h of incubation in complete DMEM, they were infected with wild-type HAdV5 (MOI 100 vp/cell) and incubated for 24 h at 37°C in 500 μL of either complete DMEM containing 10-fold IC_50_ concentration of compounds obtained in the plaque assay or the same volume of DMSO (positive control). The culture medium was then aspirated and washed with 1X with cold PBS. Later, 200 μL RIPA Lysis Buffer (1% v/v NP-40, 20 mM Tris-HCL pH 7.4, 5 mM Sodium Pyrophosphate, 5 mM EDTA) with freshly added proteinase inhibitor cocktail (Thermo Fisher Scientific) was added to each well and swirled to distribute it evenly. Cells were then scraped and collected to incubate them for 30 min on ice and finally centrifuged at 13,000 × g for 5 min at 4°C. The p53/MDM2 complex in the lysate was quantified using an ELISA assay (ENZO, Ref. ADI-960-070) according to the manufacturer’s instructions. The optical density was measured at 450 nm, and the relative levels of the p53/MDM2 complex were analyzed using a 4-parameter logistic curve in GraphPad Prism 6. This assay was performed in triplicate.

### 2.12 Knockdown of RPS14 by siRNA

An siRNA approach was used to evaluate the antiviral activity of these molecules in the absence of RPS14. According to the supplier’s protocol (Effectene Transfection Reagent, QIAGEN, Valencia, CA), 293β5 cells at 1.5 × 10^4^ cells/well were seeded in a clear bottom 96-well microplate a day before transfection. On the day of transfection, 0.1 µg siRNA (MISSION esiRNA RPS14, SigmaAldrich) was diluted in EC buffer up to 30 µL/well and 0.8 µL/well of the enhancer was added and the mix was incubated for 5 min. Effectene transfection reagent was added and the mix was incubated for 10 min to allow transfection complex formation. The cell layers were washed with 1x PBS and transfection complexes were poured over them. After 6 h of incubation, the transfection complexes were removed and the cells were infected with HAdV5-RFP (MOI 2,000 viral particles/cell) in the presence of a 10-fold IC_50_ concentration of the compounds obtained in the plaque assay or the same volume of DMSO. Standard infection was induced in the presence of the same concentrations of the compounds (performed in triplicate), and a control without siRNA. Cells, viruses, and compounds were incubated for 48 h at 37°C and 5% CO_2_. Infection, as measured by HAdV5-mediated RFP expression, was analyzed using a Typhoon 9410 imager (GE Healthcare Life Sciences) and quantified using ImageQuantTL (GE Healthcare Life Sciences).

### 2.13 HAdV/endosome colocalization

To assay the impact of rotenone on the colocalization of HAdV particles and endosomes during their entry, A549 cells (5 × 10^4^ cells/well) were seeded on coverslips, which were previously washed with 1x PBS, treated with poly-L-lysine (Merck, Darmstadt, Germany) for a minimum of 1 h and washed twice again with 1x PBS, in 12-well plates. The cells were then infected with wild-type HAdV5 (MOI 15,000 vp/cell) and incubated on ice for 1 h to synchronize the infection. Rotenone was then added at 0.05 µM followed by incubation for 60 min at 37°C. The cells were then fixed with 4% formaldehyde in 1x PBS for 20 min and permeabilized with 1x PBS/Triton X-100 0.5% for 5 min at room temperature. In addition, a blocking step was performed with PBS/BSA 10% for 1 h at room temperature. The cells were then incubated with the following primary antibodies: EE1A rabbit polyclonal IgG (Santa Cruz Biotechnology, CA, USA), a marker of early endosomes, and 9C12 hexon protein HAdV5 mouse IgG (DSHB, IA, USA) for 60 min at room temperature in a wet chamber. After rinsing with 1x PBS, the cells were incubated with the following secondary antibodies: Alexa Fluor 488 goat anti-mouse IgG (Thermo Fisher Scientific, MA, USA) and Alexa Fluor 594 goat anti-rabbit IgG (Thermo Fisher Scientific, MA, USA) for 20 min at room temperature. DAPI (Merck, Darmstadt, Germany) was added during 5 min to stain the nucleus. Finally, coverslips were fixed on the slides with mounting medium prepared using one part of 10x PBS and 9 parts of glycerol, and stored in the dark overnight at 4°C. Infected cells were observed and images were acquired using a Leica Stellaris 8 confocal microscope (Leica Microsystems, L’Hospitalet de Llobregat, Spain) with a ×63 glycerol inmersion objective (HC PL APO CS2 63x/1.30 GLYC) and LasX software (Leica). Laser lines and detectors were λ 405nm, HyD S 1 (DAPI); λ 499nm, HyD S 3 (Alexa fluor-488) y λ 590 nm, HyD S 4 (Alexa fluor 594). The Z-series was analyzed by first determining the region of interest and then measuring the fluorescence signal using ImageJ.

### 2.14 HAdV/tubulin colocalization

To evaluate the effect of rotenone on microtubular transport once the viral particles leave the endosome to address the cell nucleus, A549 cells (5 × 10^4^ cells/well) were seeded on coverslips in 12-well plates. The coverslips were previously washed with acetone, distilled water and absolute ethanol sequentially, and washed with 1x PBS, and treated with poly-L-lysine (Merck, Darmstadt, Germany) for 1 h, and washed again twice with 1x PBS. Cells were infected with wild-type HAdV5 (MOI 15,000 vp/cell) and rotenone was added at 0.05 μM. The infection was synchronized for 1 h on ice, excess virus was removed, and the cells were incubated for 90 min at 37°C and 5% CO_2_. Finally, the cells were fixed with methanol/acetone 50% for 10 min at −20°C whereupon nonspecific unions were blocked with PBS/BSA 10% for 1 h at room temperature. The cells were then incubated with hexon protein mouse monoclonal IgG (DSHB, Iowa City, USA) and anti-gamma-tubulin antibody marked by Alexa Fluor^®^ 647 (Abcam, Cambridge, UK) for 60 min at room temperature. After rinsing with 1x PBS, the cells were incubated with Alexa Fluor 488 goat anti-mouse IgG (Thermo Fisher Scientific, MA, USA) for 20 min at room temperature, and DAPI (Merck) was added for 5 min to stain the nucleus. Finally, coverslips were fixed on the slides with a mounting medium prepared from one part of 10x PBS and 9 parts of glycerol and stored in the dark overnight at 4°C. Infected cells were observed and images were acquired using a Leica Stellaris 8 confocal microscope (Leica Microsystems) with a ×63 glycerol inmersion objective (HC PL APO CS2 63x/1.30 GLYC) and LasX software (Leica). The laser lines and detectors used included λ 405nm, HyD S 1 (DAPI); λ 499nm, HyD S 3 (Alexa fluor-488) and λ 650nm, HyD S 4 (Alexa fluor 647). The Z-series was analyzed by first determining the region of interest and then measuring the fluorescence signal using ImageJ.

### 2.15 Rotenoids-CDV synergy assay

As an antiviral agent commonly used to treat HCMV and HAdV infections, CDV was selected to evaluate the potential synergistic activity of rotenone and deguelin using the Calcusyn software packet (BioSoft, Ferguson, MO, USA). This software compares the drug concentrations required in combination to generate a given effect with the drug concentration required individually to achieve the same effect. Thus, a plaque dose-response assay was performed using all the possible combinations of the three molecules (CDV, rotenone and deguelin), starting from four times the IC_50_ concentrations obtained previously for each compound and the ratio of those concentrations. CalcuSyn interpolates the drugs concentrations required in combination at the selected ratio to generate 50%, 75%, and 90% inhibition effects and compares these combined drug concentrations with those from the individual dose-response curves of the three drugs required to achieve the same inhibition. Therefore, the combination index (CI) value was reported as a quantitative estimation of the pharmacological interaction that uses the potency (IC_50_) and shape of the dose-response curve of each individual drug and their combinations. To interpret the CI values, previous publication criteria by Matthews *et al.* were followed (Matthews et al., 2017).

### 2.16 Impact on HCMV replication

To test the anti-HCMV activity of these rotenolone-related compounds, human foreskin fibroblasts (HFF; ATCC, Manassas, VA) were seeded in a 24-well plate (8.3 × 10^4^ cells/well), infected with HCMV (MOI 0.05 vp/cell), and incubated in complete DMEM in the presence of either 2-fold IC_50_ concentration of each compound obtained in the plaque assay against HAdV or the same volume of DMSO (positive control) in duplicate. The cells were incubated for 72 h at 37°C and 5% CO_2_ and HCMV DNA was purified from the cell lysate using E.Z.N.A Tissue DNA Kit (Omega Biotek, Norcross, GA, USA) following the manufacturer’s instructions. The qPCR primers, conditions, and protocols were the same as previously reported (Mazzotta et al., 2020b).

### 2.17 Statistical analyses

One-way ANOVA (Dunnett’s method) was performed using the GraphPad Prism 6 software. A *p*-value less than 0.05 was considered statistically significant. Statistically significant values were marked with asterisks in the graphs, and their numbers indicate the level of significance (*p ≤ 0.05, **p ≤ 0.01, ****p ≤ 0.0001).

## 3 Results

### 3.1 Anti-adenovirus activity

By exploiting the CM1407 Action’s chemical library, we screened 1,233 molecules at an initial concentration of 50 µM to assess their anti-HAdV activity. To this end, human A549 epithelial cells infected with replication-defective viruses containing a CMV promoter-driven enhanced green fluorescent protein (eGFP) reporter gene cassette (2,000 vp/cell) were used. Among these, we initially identified 18 molecules that reduced HAdV5-GFP transgene expression by more than 50%. Rotenolone (Figure 1) exhibited 100% inhibition of HAdV5-GFP transgene expression and was selected for the evaluation of its effect on cellular viability and to further characterize its anti-HAdV activity. In addition, four other molecules, rotenone, deguelin, millettone, and tephrosin, whose structures are closely related to rotenolone, were included in our study (Figure 1).

Rotenolone and all related molecules reproducibly inhibited HAdV5 infection in a dose-dependent manner and displayed 100% inhibition of plaque formation at low nanomolar concentrations (except millettone, which required higher concentrations) (Supplementary Figure S1). While millettone inhibited more than 98% the plaque formation at 10 μM, the other compounds inhibited more than 94% HAdV5-GFP plaque formation at nanomolar concentrations, reaching an inhibition percentage of 67.8% at a concentrations as low as 4 nM in the case of rotenone.

The IC_50_ values of all rotenolone-related molecules against HAdV5-GFP are summarized in Table 1. Millettone displayed the highest IC_50_ value (4.6 µM), whereas rotenone exhibited the lowest (3.9 nM), 10 times lower than the IC_50_ observed for the other three molecules. The four molecules presented variable levels of cytotoxicity with CC_50_ values ranging from 0.6 µM to 109.4 µM, which translated into selectivity indices (SIs) ranging from 23.8 to 2,429.3 for millettone and deguelin, respectively (Table 1). Next, we examined the effect of the molecules with SI >100 on virus replication using a virus burst assay to measure virus particles production. As summarized in Supplementary Table S1, treatment with a 10-fold concentration of the IC_50_ of each compound was associated with a significant reductions in HAdV5 yield. Treatment with rotenolone, rotenone, and tephrosin reduced the viral yield by 2,700 to 13,000-fold, while deguelin displayed a 270-fold reduction.

**TABLE 1:** IC_50_, CC_50_, and SI for rotenolone-related compounds against HAdV5. The results represent means ± SD of duplicate samples from two independent experiments.

Compound	IC_50_ plaque assay (µM)	CC_50_ (µM)	Selectivity index
Rotenolone	0.03 ± 0.00	17.1 ± 0.4	569.0
Rotenone	0.0039 ± 0.0001	0.6 ± 0.2	164.1
Millettone	4.6 ± 0.54	109.4 ± 2.2	23.8
Deguelin	0.04 ± 0.01	97.2 ± 0.1	2,429.3
Tephrosin	0.07 ± 0.03	37.7 ± 0.6	538.9

To assess whether the activity of the molecule with the lowest IC_50_ (rotenone) and that with the highest SI (deguelin) improved in combination with CDV, a combination study was conducted based on the Chou−Talalay method for drug combinations using CalcuSyn software (Chou and Talalay, 1984; Matthews et al., 2017). A constant ratio for each combination was selected based on the IC_50_ value of each molecule. The data for both combinations conformed well with the principle of mass law (r) (Table 2). The combination of rotenone with CDV was classified as synergistic at ED_50_ and moderately synergistic at ED_75_ and ED_90_. The deguelin-CDV combination was classified as synergistic at ED_75_ and ED_90_ but strongly synergistic at ED_50_.

**TABLE 2:** Combination index (CI) obtained using CalcuSyn software for both cidofovir (CDV)+rotenone and cidofovir+deguelin combinations.

Compound	ED_50_	ED_75_	ED_90_	r
CDV+Rotenone	0.678	0.704	0.731	0.93
CDV+Deguelin	0.252	0.329	0.443	0.86

Our infection assays indicated that although treatment with these molecules blocked HAdV5 infection, it did not provide any indication of the mechanism of inhibition. Because the HAdV replicative cycle is a highly coordinated multistage process, interference at any of these steps would ultimately result in a reduction in virus yield. Therefore, our next objective was to determine the specific steps in the HAdV infection pathway targeted by these molecules.

### 3.2 Impact of rotenolone-related compounds on p53/MDM2 interaction

Because of the high similarities in the chemical structures of these rotenoids with that of emetine, we hypothesized that they could demonstrate the same antiviral mechanism of action as previously reported against HCMV for emetine, where emetine disrupts the HCMV-induced MDM2-p53 interaction (Mukhopadhyay et al., 2016). In response to various genotoxic stresses, p53 transactivates several genes by binding to specific DNA sequences (Kato et al., 2003). MDM2 is one of the main regulators of this tumor suppressor protein. Under normal conditions, MDM2 induces proteasomal degradation of p53 via polyubiquitination and inhibition of transactivity of p53, however, upon DNA damage, MDM2 enhances p53 translation (Hernández-Monge et al., 2016). The interaction between MDM2 and p53 was favoured upon HCMV infection and was disrupted by emetine in high-density cells, but not in low-density cells.

As shown in Supplementary Figure S2A, when we evaluated the concentration of p53/MDM2 complexes in cell cultures infected with HAdV, only rotenolone presented a significant difference when comparing concentrations at high and low cell densities, with the highest concentrations observed in plates with low cell density (**p ≤ 0.01). In contrast to HAdV, emetine dramatically reduced the concentration of p53/MDM2 complexes, but there were no differences based on the cell density tested.

Additionally, Mukhopadhyay et al. (2016) reported the ribosomal protein S14 (RPS14) as a target of emetine, and we investigated the anti-HAdV activity of these rotenolone-related molecules in RPS14 knockdown cells. Once we confirmed the decrease in RPS14 expression by Western blotting, both at 24 h and 48 h post infection (p.i.), we evaluated the anti-HAdV activity of these molecules. As shown in Supplementary Figure S2B, all of the assayed molecules, including emetine, reduced the HAdV-mediated RFP expression when no RPS14 was available, as they did when tested without the RPS14 knockdown (emetine exhibited an IC_50_ of 85 ± 19 nM and a CC_50_ of 6.17 ± 0.04 µM). These results suggest that the mechanism of action of emetine and rotenolone-related molecules against HAdV involves a different route than that against HCMV, at least partially.

### 3.3 Impact of rotenolone-related compounds on HAdV DNA replication

Once a mechanism of action similar to that of emetine was ruled out, we focused our efforts on identifying the potential targets of these molecules to block HAdV infection. Once the partially uncoated particles reach the nuclear membrane of their host cell, HAdV genomes, together with HAdV protein VII, are translocated into the nucleus, where they undergo transcription of early mRNA, followed by DNA replication and late mRNA synthesis (Mangel and San Martin, 2014; Charman et al., 2019). Thus, we investigated whether any of the selected compounds blocked *de novo* synthesis of HAdV DNA copies compared with a positive control under the same experimental conditions; we observed that only rotenone and deguelin displayed a significant decrease in the HAdV DNA copy number (Supplementary Figure S3A). A reasonable explanation for this decrease in HAdV DNA replication in the presence of these compounds is a decrease in the total number of HAdV DNA copies that reached the nucleus. To confirm this hypothesis, we assayed if these natural compounds can block HAdV genome access to the nucleus by quantifying the HAdV genomes isolated from the nucleus after 45 min of synchronized infection. As shown in Supplementary Figure S3B, only rotenone significantly blocked the accessibility of HAdV genomes to the nucleus (*p* ≤ 0.01).

### 3.4 Time of addition curve

**FIGURE 2:**
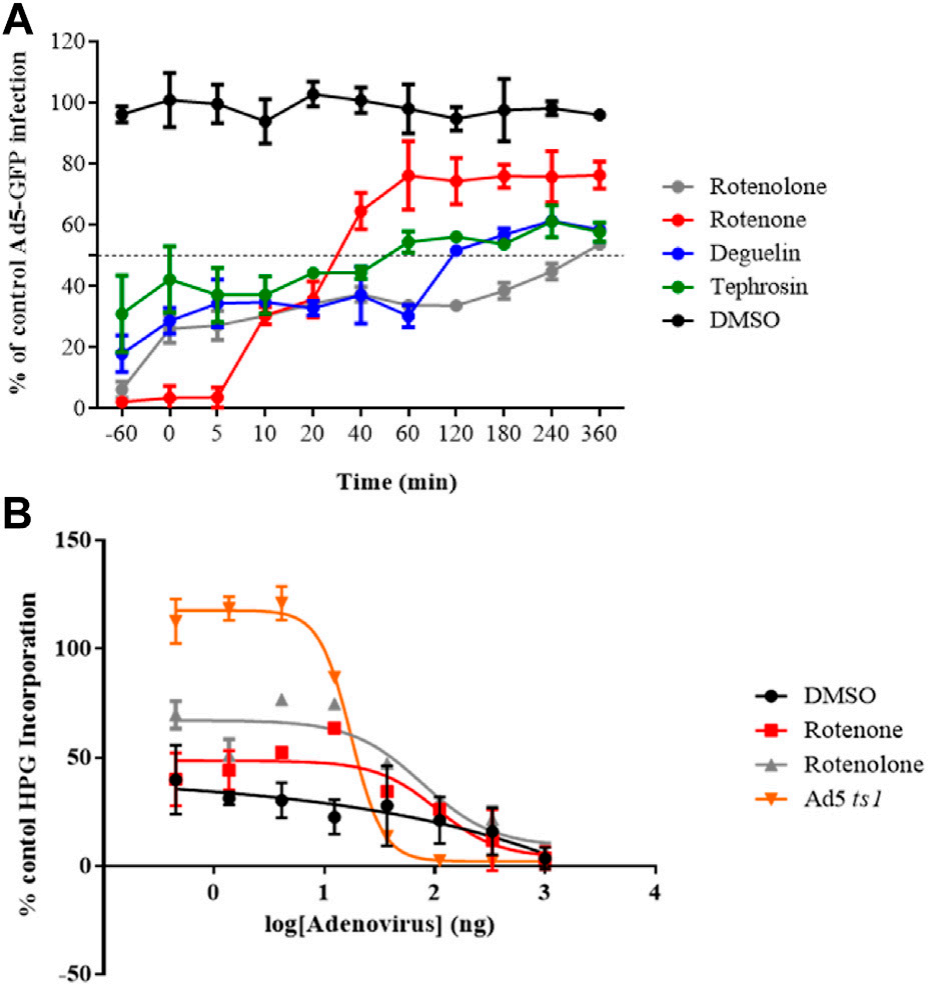
**(A)** Time of addition assay. Effect of selected molecules on HAdV infection at different time points at 10-fold the IC_50_ concentration obtained in the plaque assay for each of them. The DMSO control is a negative control with cells infected at the same MOI and treated with DMSO at the same time points but in the absence of drugs. Results represent means ± SD of duplicate samples from independent experiments. **(B)** Impact on HAdV endosomolysis. Rotenone and rotenolone did not attenuate HAdV-mediated endosomolysi in our α-sarcin assay, as describe in Methods. Data shows the percentages of L-homopropargylglycine (HPG) incorporation. Results represent means ± SD of triplicate assays.

To further understand the inhibition mechanism of these molecules, we assayed if the time of addition of these compounds influenced their ability to block HAdV infection at specific stages of the replicative cycle. HAdV species C has been reported to be internalized within 5 min of binding, escape the endosome after 15 min, and attach to the nuclear pore complex after 35–45 min (Greber et al., 1993). We observed that all compounds exhibited a slight time-dependent decrease in inhibitory activity, except rotenone, which exhibited a sharp loss of inhibitory activity (more than 25%) when added at 10 min p.i. and more than 60% at 40 min p.i (Figure 2A). These results imply that rotenone inhibited an early step in viral entry after cell attachment. In addition, the targets of rotenolone, degueline and tephrosin appear to be associated with the later stages of the HAdV replicative cycle. This was supported by the low percentage of HAdV genomes that reached the cell nucleus, indicating an HAdV-mediated endosome disruption and escape to the cytoplasm as potential routes impacted by rotenone.

### 3.5 Impact of rotenone on HAdV endosomolysis

To confirm whether rotenone is involved in the blocking of HAdV-mediated endosome disruption, a ribotoxin co-delivery assay was performed. In this assay, the HAdV-mediated endosome lysis is indicated by reduced cellular protein synthesis, measured by the amino acid analogue of methionine L-homopropargylglycine (HPG) incorporated into the host proteins upon HAdV escape from the endosome (Marrugal-Lorenzo et al., 2019). Rotenolone was included as a negative control because it did not block the accessibility of HAdV to the nucleus. As shown in Figure 2B, cells treated with either the vehicle (DMSO), rotenone, or rotenolone exhibited low rates of HPG incorporation (approximately 30%) compared with a control without α-sarcin, independent of the virus concentration. In contrast, the entry-defective AdV2*ts*1 mutant, which contains a mutation in the protease gene and fails to penetrate cell endosomes, does not mediate endosome lysis at low viral concentration. This result is in agreement with our results on HAdV colocalization with early endosomes, which displayed similar patterns of colocalization at 60-min p.i., exhibiting a scattered distribution above the cellular nucleus in the presence and absence of rotenone (Supplementary Figure S4). This suggests that the target for rotenone must be located at a later stage, probably during the microtubular transport to the nuclear membrane.

Before evaluating the potential interference of rotenone with the cytoplasmic transport of the viral particles to the nucleus, we assayed whether the inhibitory activity of rotenone was associated with a direct interaction with HAdV particles. HAdV-RFP was incubated with rotenone (0.1 µM) in duplicates for 45 min on ice to allow rotenone interact with any potential viral particle structure. These treated virions were then added to A549 cells and incubated for 1 h on ice, to allow the HAdV particles to attach to the cells. After this incubation, the cells were washed with 2x PBS to remove excess virus, and the medium was replaced with medium with another one containing the same concentration of rotenone in one case and without rotenone in another, and incubated for another 48 h. Figure 4A shows no differences in the level of RFP inhibition between the cells incubated with or without rotenone. These results suggest that there is a direct interaction between rotenone and the HAdV particles.

### 3.6 Impact of rotenone on HAdV microtubule association

**FIGURE 3:**
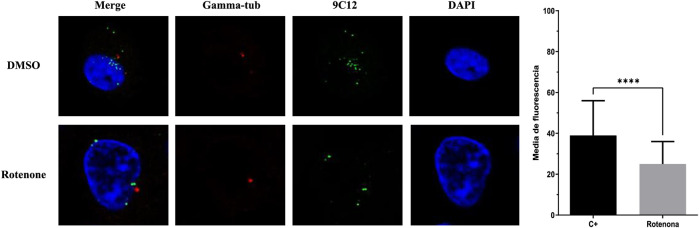
Rotenone impact on HAdV colocalization with tubulin. Representative cells at 90 min p.i. with tubulin labelled with Alexa Fluor^®^ 647 anti-gamma-tubulin antibody, HAdV5 labelled with 9C12 hexon protein HAdV5 IgG (secondary antibody Alexa Fluor 488, green) and cellular nucleus labelled with DAPI (blue). Images are Z-slices from a sequential Z-serie. The graph depicts the mean fluorescence of the gamma-tubulin within the region enclosed by the virus. Statistical significance was pointed out with asterisks in graph (****p ≤ 0.01).

To further characterize the steps in the HAdV infection route blocked by rotenone, A549 cells were infected with HAdV5 in the presence and absence of rotenone, and the distribution of viral particles was analyzed with respect to the nucleus and the microtubule network using fluorescence microscopy 90 min after synchronized infection. After escaping from the endosome, HAdV particles associate with microtubules and migrate to the nuclear pore complex (NPC), through which the genome is transported into the nucleus. In the absence of rotenone, HAdV particles localize to the apical region of the nucleus in the so-called microtubule-organizing centers (MTOC). However, in the presence of rotenone, altered transport of HAdV to the MTOC was observed, which resulted in a lower level of colocalization of HAdV particles with the tubulin (Figure 3).

### 3.7 Impact of these rotenolone-related molecules on HCMV replication

**FIGURE 4:**
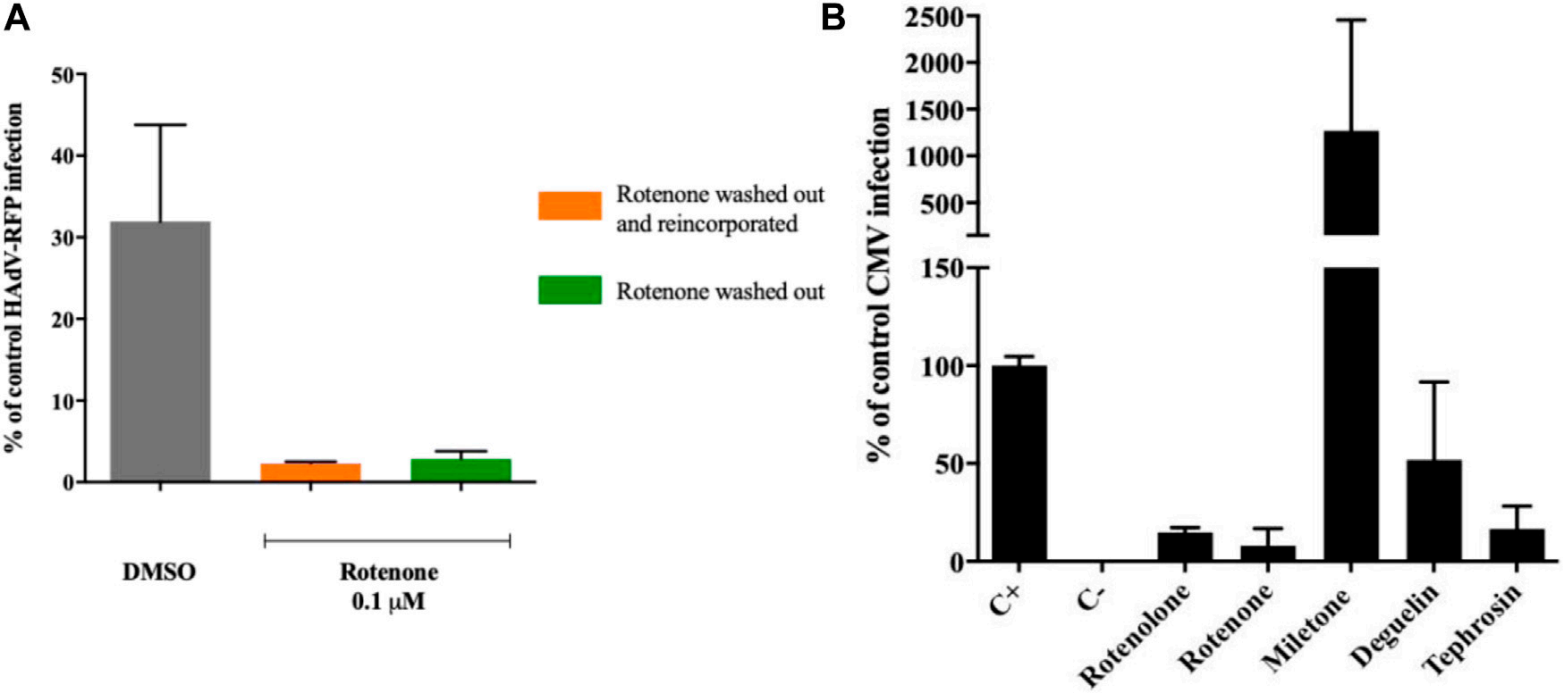
**(A)** Rotenone direct interaction with HAdV virions. Rotenone keeps its antiviral activity after HAdV attachment to its cellular receptors and after removal from the media. **(B)** Impact of rotenolone-related compounds on HCMV replication. Quantification of HCMV DNA copies 72 h post-infection by a quantitative PCR assay. Results represent means ± SD of triplicate assays.

Finally, as a proof-of-concept for the potential broad antiviral activity of these rotenolone-related molecules, their antiviral activity was assayed against HCMV using an assay to measure the HCMV replication efficiency in their presence by qPCR. As illustrated in Figure 4B, millettone not only did not inhibit HCMV replication, but increased it by more than 10 times compared with the control treated with DMSO. Deguelin exhibited a 50% inhibition of HCMV replication whereas rotenolone, tephrosine, and rotenone displayed the highest levels of inhibition with inhibition (85.2%, 83.4%, and 92.1%, respectively).

## 4 Discussion

In the current study, we identified four rotenolone-related compounds that significantly inhibited HAdV and HCMV infections at low nanomolar and sub-micromolar concentrations and high SI. Our results suggest that the antiviral mechanism of action of rotenone involves interfering with the transport of the viral particles from the endosome to the cell nucleus, whereas that of rotenolone, deguelin, and tephrosin appears to be related to later stages in the HAdV replicative cycle.

The SARS-CoV-2 pandemic has exposed the scarcity of antiviral drugs. In this context, the lack of any approved antiviral drug to treat HAdV infections exposes another unfulfilled medical need that must be addressed because of its high clinical impact (Martinez et al., 2015; Aguilar-Guisado et al., 2020).

Screening for antiviral molecules from natural products has several advantages over chemically synthetized compounds. The large structural diversity and complexity of natural products allow them to interfere with different biological pathways (Martinez et al., 2015). This is the case for flavonoids, secondary metabolites produced by plants with reported antiviral activity against respiratory syncytial virus, HCMV, hepatitis C virus, herpes simplex virus types 1 and 2, Newcastle virus, and chikungunya virus (Takatsuki et al., 1969; Arakawa et al., 2009; Zakaryan et al., 2017; Nukui et al., 2018; Liao et al., 2020); similarly, glycans extracted from marine organisms have exhibited antiviral effects against RSV, HSV-1, HSV-2, dengue virus, human immunodeficiency, virus and HCMV (Grice and Mariottini, 2018; Zoepfl et al., 2023).

The anti-HAdV activity observed in the molecules in this study was better than that of other natural and synthetic compounds previously identified by our group (Sanchez-Cespedes et al., 2016; Marrugal-Lorenzo et al., 2018; Marrugal-Lorenzo et al., 2019; Mazzotta et al., 2020a; Xu et al., 2020a; Mazzotta et al., 2020b; Xu et al., 2020b; Pech-Puch et al., 2020). All rotenoids exhibited the greatest antiviral activity at nanomolar concentrations as low as 4.625 nM and with SI > 100, indicating their specificity.

As for their mechanism of action, the results obtained from the evaluation of their impact on HAdV DNA replication and from the time curve for their activity suggest that rotenolone, deguelin and tephrosin exert their effect in the late stages of the HAdV replicative cycle, after DNA replication; a similar observation has been previously described for a series of synthetic molecules whose structure was inspired by niclosamide, an anthelmintic drug approved by the health authorities, which has been demonstrated to have broad-spectrum antiviral activity. (Xu et al., 2020a; Xu et al., 2020b). In contrast, rotenone appeared to act in the early stages of HAdV entry into the cell.

Our results indicate that non among emetine, rotenone, deguelin and tephrosin alter the concentrations of the p53/MDM2 complexes when compared at low and high cell densities. In addition, all of the molecules tested, especially emetine, demonstrated antiviral activity even when RPS14, the specific predicted target for emetine, was silenced. Only rotenolone presented a significant difference when comparing concentrations at high and low cell densities, with the highest concentrations observed in plates with low cell density (**p ≤ 0.01). Considered together, these results suggest the existence of an alternative mechanism of action for emetine and rotenolone-related molecules against HAdV.

Although other rotenolone-related compounds also displayed a progressive loss of antiviral activity over time, rotenone was the only molecule that exhibited a significant loss of activity early during HAdV infection, similar to compound 16, a previously identified niclosamide derivative with high anti-HAdV activity (Xu et al., 2021). This result implies that this molecule targets one of the early stages in the HAdV replicative cycle, that is, from the attachment and internalization of the viral particles that occur immediately after the first contact with the cells, to the endosomal escape or viral transport to the nuclear membrane (Xu et al., 2021). Our work indicates that the antiviral activity of rotenone involves a direct interaction with the viral particle, which would fit any of the mentioned options. Our α-sarcin co-delivery physiological assay revealed that rotenone exhibited an opposite behavior compared to the entry-defective HAdV2*ts1* mutant, which contains a mutation in the protease gene and fails to penetrate cell endosomes. This result is consistent with previous results obtained with mifepristone, a synthetic steroid related to progesterone and glucocorticoids, where it interfered with the transport of HAdV DNA from the endosome to the cell nucleus (Marrugal-Lorenzo et al., 2018). Thus, we suggest that the mechanism for the inhibition of rotenone is likely related to the blockage of the transport of partially uncoated HAdV particles to the nuclear membrane through the microtubular network. This was also supported by our co-localization assays that indicated a similar pattern of HAdV particles co-localization with endosomes at 60 min p.i. when compared with a control with DMSO, unlike, for example, for α-defensin HD5 (Smith and Nemerow, 2008).

At this point, similar to when we characterized the antiviral activity of mifepristone against HAdV (Marrugal-Lorenzo et al., 2018), we considered the possibility that rotenone acts by targeting HAdV shifting along microtubules, thus preventing the translocation of viral particles to the MTOC (Strunze et al., 2005). Our results suggest that rotenone induces an altered transport of the viral particles, as evidenced by the lower colocalization of HAdV particles with the tubulin, which may preclude HAdV NPC association and genome translocation into the nucleus. This observation is in agreement with the proposed mechanism of rotenone to inhibit mammalian cell proliferation at µM concentrations, including the inhibition of microtubule polymerization and the interference with the microtubule assembly dynamics (Srivastava and Panda, 2007).

## 5 Conclusion

Rotenone, rotenolone, diguelin and tephrosin have been proven to exert high antiviral activity against HAdV and HCMV at nanomolar and submicromolar concentrations and exhibit high SI. Unlike rotenolone, diguelin and tephrosin, which appear to act during the late stages of the HAdV replicative cycle, the mechanism of action of rotenone appears to be related to its capacity to alter microtubular polymerization, preventing HAdV particles from reaching the nuclear membrane of the host cell. Although further characterization of the mechanisms of action of these rotenoids is still required, they could be potentially strong hit candidates for the development of a new family of broad-spectrum antiviral drugs.

## Data Availability

The raw data that support the findings of this study are available from the corresponding authors (DQ/JSC) upon reasonable request.
