# Supplementary material for: Inhibition of adenovirus transport from the endosome to the cell nucleus by rotenone
Source: Front Pharmacol. 2024 Jan 11;14:1293296. doi: 10.3389/fphar.2023.1293296 (PMC10808720; doi:10.3389/fphar.2023.1293296)
Supplement: Supplementary file 1 [file DataSheet1.pdf]

Supplementary material

Inhibition of adenovirus transport from the endosome to the cell nucleus by rotenone

María Balsera-Manzanero[a,b#], Francesca Ghirga[c#], Ana Ruiz-Molina[a,b#], Mattia Mori[d], Jerónimo Pachón[b,e], Bruno Botta[c], Elisa Cordero[a,b,e,f], Deborah Quaglio[c*] and Javier Sánchez-Céspedes[a,b,f*]

[a]Division of Infectious Diseases, Microbiology and Parasitology, Virgen del Rocío University Hospital, Seville, Spain.

[b]Institute of Biomedicine of Seville (IBiS), Virgen del Rocío University Hospital/CSIC/University of Seville, Seville, Spain.

[c]Department of Chemistry and Technology of Drugs, "Department of Excellence 2018−2022", Sapienza University of Rome, 00185 Rome, Italy.

[d]Department of Biotechnology, Chemistry and Pharmacy, "Department of Excellence 2018−2022", University of Siena, 53100 Siena, Italy.

[e]Department of Medicine, University of Seville, E-41009 Seville, Spain.

[f]CIBERINFEC, ISCIII - CIBER de Enfermedades Infecciosas, Instituto de Salud Carlos III, Madrid, Spain.

Figure S1. Inhibitory activity of rotenolone (**a**), rotenone (**b**), millettone (**c**), deguelin (**d**) and tephrosin (**e**). Dose-dependent activity against HAdV5 in a plaque assay at low MOI. Results represent means ± SD of duplicate samples from independent experiments.

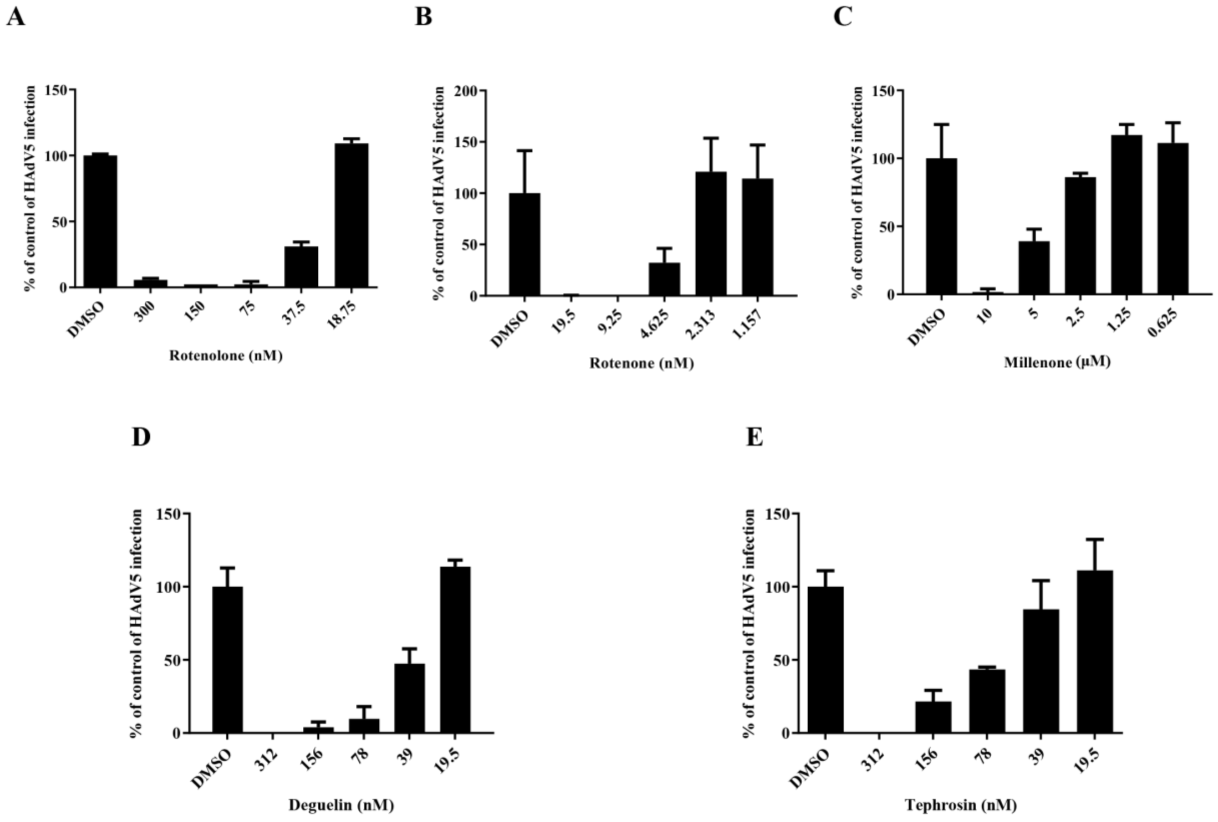

Table S1. Quantification of virus yield in the presence of rotenolone-related compounds against HAdV5. Virus production in presence and absence of rotenolone-related compounds was calculated in a burst assay followed by an endpoint dilution assay [1]. The results represent means ± SD of duplicate samples from two independent experiments.

Compound	Virus Yield Fold-reduction*
**Rotenolone**	1.3E+04 ± 4582.5
**Rotenone**	6.3E+03 ± 1636.2
**Millettone**	ND
**Deguelin**	2.7E+02 ± 19.4
**Tephrosin**	2.7E+03 ± 987.3

*TCID50/ml compound *vs.* DMSO; ND: not determined

Figure S2. (A) Impact of rotenolone, rotenone, deguelin, and tephrosin on *p53/MDM2* interaction and HAdV infection at high and low cell culture densities. Positive control are non-infected cells and treated with DMSO (vehicle) and negative control are cells where DMSO has been added to cells infected at the same MOI in the absence of compounds. Results represent means ± SD of duplicate samples from independent experiments. Statistical significance was pointed out with asterisks in graph (**p ≤ 0.01). (B) Impact of RPS14 knockdown on the anti-HAdV activity of rotenolone-related compounds. Results represent means ± SD of duplicate samples from independent experiments. Statistical significance was pointed out with asterisks in graph (****p ≤ 0.01).

A

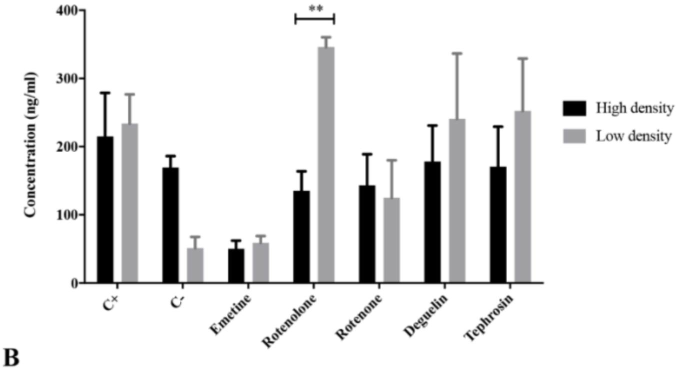

B

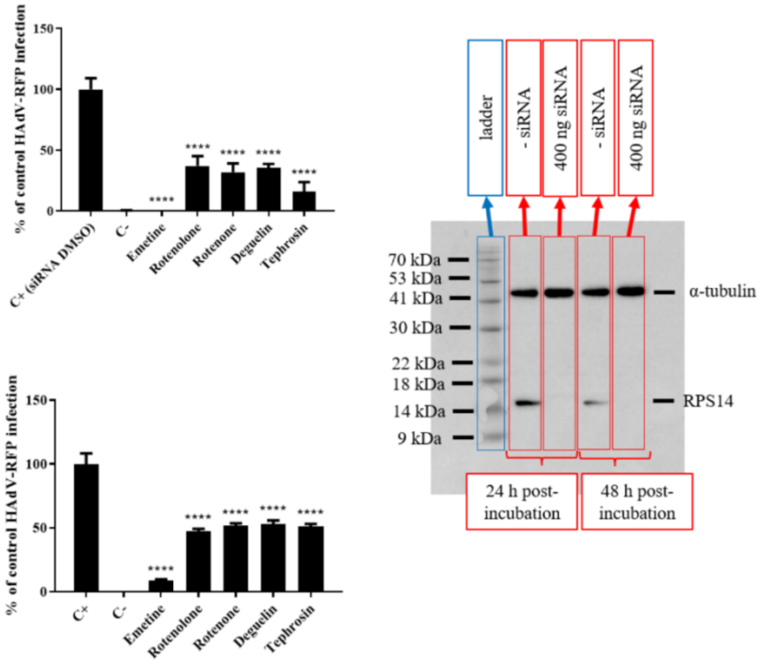

Figure S3. Impact of rotenolone, rotenone, deguelin, and tephrosin on HAdV replication (**A**) and HAdV accessibility to the nucleus cell in presence of the same compounds (**B**). The DMSO control is a negative control with cells infected at the same MOI in the absence of compounds. Results represent means ± SD of duplicate samples from independent experiments. Statistical significance was pointed out with asterisks in graph (**p ≤ 0.01).

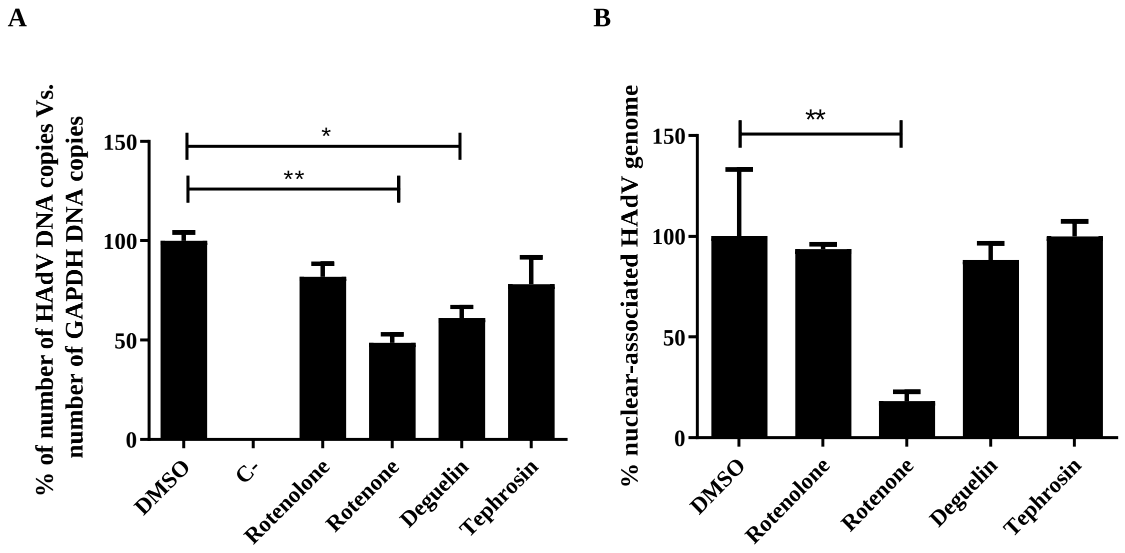

Figure S4. Rotenone impact on HAdV colocalization with endosomes. Representative cells at 60 min p.i. with endosome labelled with EE1A rabbit polyclonal IgG (secondary antibody Alexa Fluor 594, red), HAdV5 labelled with 9C12 hexon protein HAdV5 mouse IgG (secondary antibody Alexa Fluor 488, green) and cellular nucleus labelled with DAPI (blue). Images are Z-slices from a sequential Z-serie. The graph depicts the mean fluorescence of the virus within the region enclosed by the endosomes. No statistical significance observed in graph.

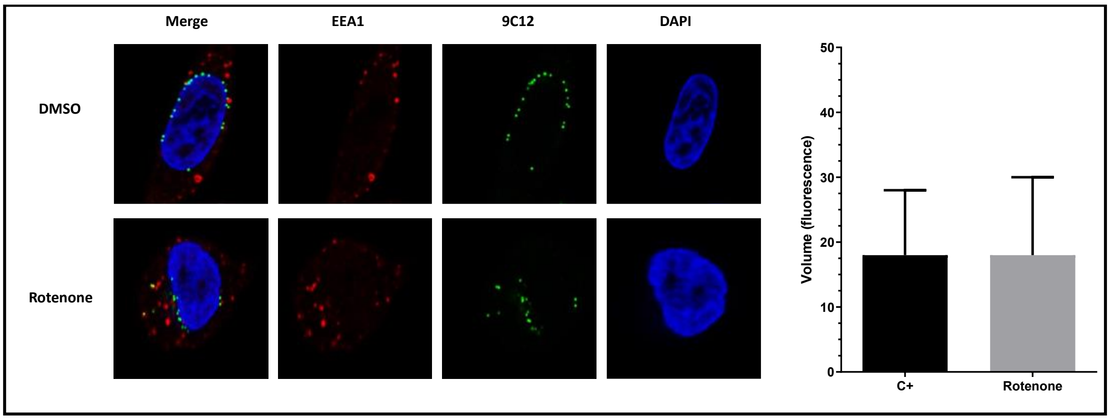

References

[1] Reed LJ, Muench, H. A simple method of stimating fifty percent endpoints. American Journal of Hygiene. 1938;27:4.
